# HCAR1-mediated lactate signaling modulates motor behavior and regulates spontaneous firing in Purkinje cells

**DOI:** 10.1016/j.isci.2026.116930

**Published:** 2026-08-03

**Authors:** Francesca Binda, Lara Buscemi, Nicolas Gurtner, Lorenz Hirt, Jean-Yves Chatton

**Affiliations:** 1 Department of Fundamental Neurosciences, University of Lausanne, 1005 Lausanne, Switzerland; 2 Stroke Laboratory, Neurology Service, Department of Clinical Neurosciences, Lausanne University Hospital Centre and University of Lausanne, 1011 Lausanne, Switzerland; 3 Cellular Imaging Facility, University of Lausanne, 1005 Lausanne, Switzerland

**Keywords:** lactate, HCAR1, cerebellum, Purkinje cells, HCN, spontaneous firing, hydroxycarboxylic acid receptor 1, motor learning, glia

## Abstract

Lactate modulates several phenomena ranging from gene transcription to plasticity. Through its specific GPCR receptor HCAR1, lactate acts as a signaling molecule modulating neuronal excitability. Silencing lactate signaling promotes anxiety-like behavior and worsens epileptic seizures, highlighting the central role of HCAR1 in brain function.

HCAR1 expression in the cerebellum supports the role of the receptor in motor coordination. Early experimental evidence suggesting HCAR1 expression in cerebellar Purkinje cells (PCs) together with its ability to modulate neuronal excitability qualifies the receptor as a possible modulator of PC activity with important implications for cerebellar-mediated diseases.

This hypothesis was investigated using behavioral analysis and *ex vivo* electrophysiology that showed gait instability and motor learning defects in HCAR1 knockout mice associated with increased PC firing. We identified HCN channels as the downstream target of HCAR1.

We conclude that lactate signaling is a modulator of PC excitability and revealed the HCAR1 role in motor behavior.

## Highlights

• HCAR1-mediated lactate signaling is required for motor stability and motor learning
• HCAR1 is a novel modulator of spontaneous Purkinje cell firing
• HCAR1-mediated lactate signaling modulates HCN channels in Purkinje cells

## Graphical abstract

**Figure:**
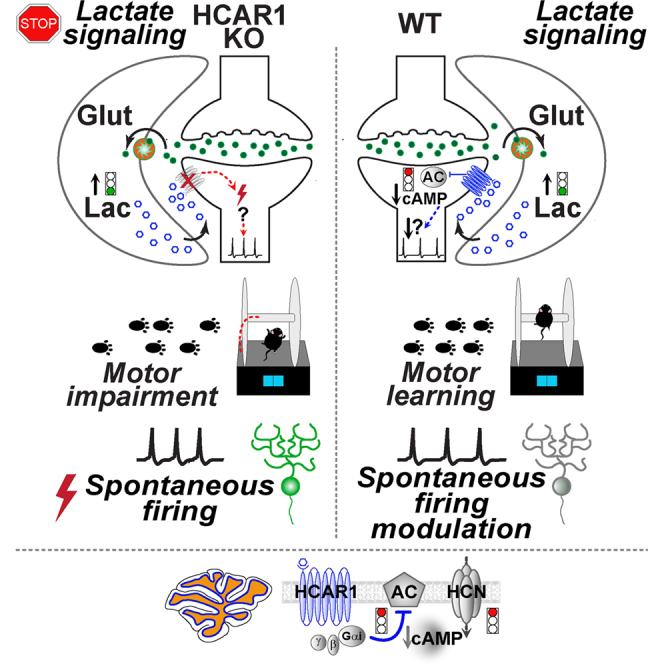

## Teaser

Systems neuroscience; Cellular neuroscience

## Introduction

Lactate was long considered merely a waste product of metabolism and its presence only viewed as a hallmark of incomplete respiration and oxygen deprivation. This view drastically changed when the ability of neurons to use lactate as an energy substrate was discovered and the astrocyte-neuron lactate shuttle hypothesis was proposed.^1^^,^^2^ Glial cells and the vascular system provide lactate to neurons that use monocarboxylate transporters (MCTs) for its uptake.^3^ While the interstitial brain lactate concentration lies in the low millimolar range at rest,^4^^,^^5^ a 2-fold increase can be observed during synaptic activity.^6^ Furthermore, intense physical exercise raises plasma lactate concentration up to 10–20 mM, favoring lactate entrance into the brain by crossing the blood-brain barrier.^7^

Strong evidence supports the shuttling of energy-rich substrates such as lactate between glia and neurons in the cerebellum, where transport and metabolism of glucose are preferentially attributed to Bergmann glial (BG) over Purkinje cells (PCs).^8^^,^^9^ Cerebellar interstitial lactate is increased by 30% during climbing fiber (CF) stimulation, whereas glucose remains constant.^10^ The expression of transporters responsible for the release of lactate by BG (MCT4) and its uptake by PCs (MCT2)^11^ further supports the existence of a lactate shuttle in the healthy cerebellum. Interestingly, in humans, NMR spectroscopy in patients with ataxia revealed elevated cerebellar lactate levels possibly linked to mitochondrial disorders.^12^

It became evident that lactate also serves as a signaling molecule^13^ when its specific receptor was identified. Lactate activates a Gi-protein-coupled receptor (GPCR) known as hydroxycarboxylic acid receptor 1 (HCAR1, formerly GPR81), initially identified in adipocytes.^14^^,^^15^

HCAR1 is expressed in neurons,^16^^,^^17^ and its activation by lactate and by non-metabolized agonists leads to a down-modulation of neuronal activity in mouse, rat, and human brain tissue.^16^^,^^18^^,^^19^ HCAR1 is currently the only identified plasma membrane receptor for lactate, and its detailed structure was recently resolved by cryo-EM.^20^ HCAR1 is a Gαi-coupled receptor.^21^ The signaling cascade induced by HCAR1 activation in neurons leads to the downmodulation of the cyclic AMP-dependent protein kinase A (PKA) through the inhibition of the adenylyl cyclase (AC).^18^ Lactate and HCAR1 play an important role in epilepsy by modulating neuronal circuit excitability and limiting seizure spreading.^22^^,^^23^ Also, silencing the receptor promotes anxiety-like behavior.^24^

Within the CNS, HCAR1 is expressed in several areas (i.e., hippocampus, cortex, and cerebellum) with enrichment in the cerebellum, where it associates with PCs,^19^ the sole output of the cerebellar cortex. Whereas in primary cortical neurons^16^^,^^18^ and in hippocampal neurons *in situ*^19^ HCAR1 reduces spontaneous neuronal firing and excitability,^16^^,^^18^^,^^19^^,^^25^^,^^26^ its role in cerebellar physiology and motor coordination remains unknown.

To address this knowledge gap, we examined the functional role of HCAR1-mediated signaling in cerebellar PC activity and motor behavior. To overcome the absence of specific HCAR1 antagonists, we took advantage of the murine HCAR1 knockout line,^27^ and we characterized the downstream effects caused by the absence of the receptor by performing behavior analysis and *ex vivo* electrophysiological recordings. Our study shows that HCAR1-deficient mice have gait instability and motor learning deficits compatible with a cerebellar origin of these defects; at the cortical level, alterations of spontaneous PC firing were also identified. We provide evidence supporting HCAR1-mediated downmodulation of the cAMP-sensitive hyperpolarization and cyclic nucleotide-gated channels (HCNs) as the mechanism that mediates PC firing modulation.

## Results

### Motor behavior is altered in HCAR1 knockout mice

**Figure 1:**
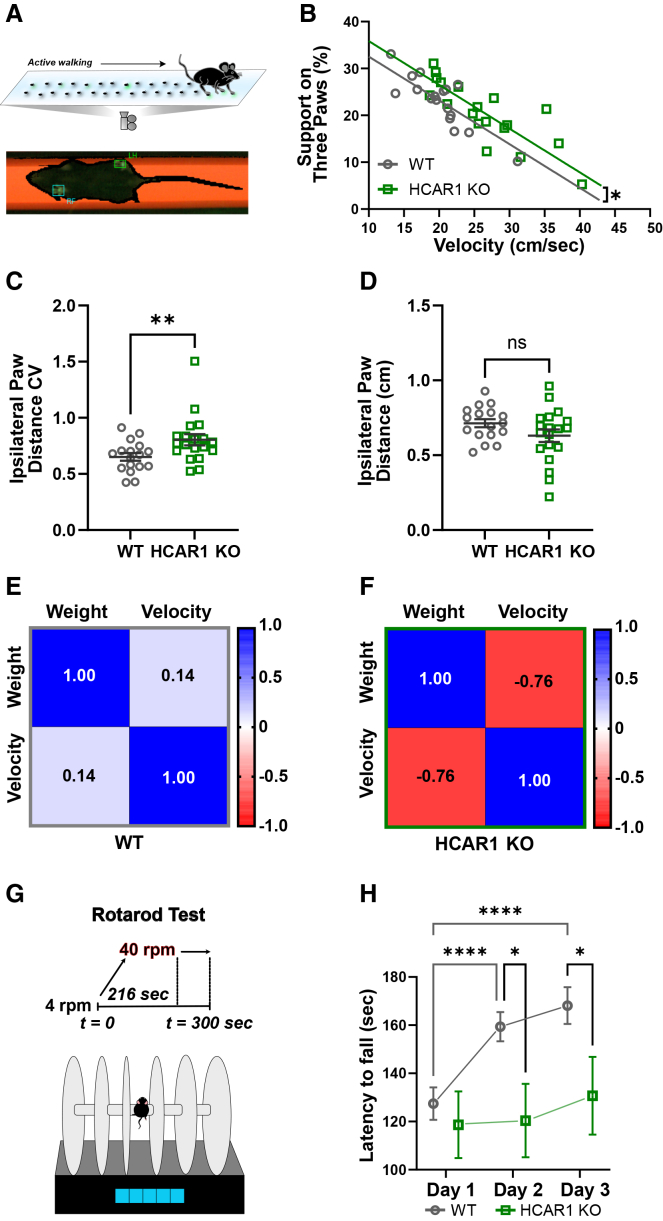
HCAR1 KO mice exhibit gait instability and motor learning impairment (A) Schematic drawing of the Catwalk test. (B) Three-paws support vs. velocity plot (linear fitting: WT, gray line, KO green line); ∗ = *p* < 0.05, multiple linear regression, WT: *N* = 17, KO: *N* = 19. (C) Scatter plot showing the coefficient of variation (CV) of the ipsilateral paw distance in WT and KO mice; mean ± SEM, ∗∗ = *p* < 0.01, Mann-Whitney test, WT: *N* = 17, KO: *N* = 19. (D) Scatter plot showing the ipsilateral paw distance quantified for WT and KO mice; mean ± SEM, n.s = *p* > 0.05, unpaired *t* test, WT: *N* = 17, KO: *N* = 19. (E) Correlation matrix with Pearson’s r values showing the correlation between weight and velocity in WT mice. (F) Correlation matrix with Pearson’s r values showing the correlation between weight and velocity in KO mice. (G) Schematic drawing of the rotarod test and protocol used. (H) Scatter plot showing the latency to fall from the rotating rod quantified over the three days of testing for WT and KO mice; mean ± SEM. ∗ = *p* < 0.05 and ∗∗∗∗ = *p* < 0.0001, two-way RM ANOVA, WT: *N* = 34, KO; *N* = 9. Also see Figure S1 and Table S1.

We previously identified the cerebellum as an area enriched in HCAR1-encoding mRNA^19^ supporting a role of the receptor in modulating cerebellar activity. Since the cerebellum is central to motor coordination, we performed gait analysis in freely walking WT and HCAR1 KO (KO) mice to identify abnormalities caused by the absence of the receptor (Figure 1A). KO mice traversed the walkway at higher velocity compared to WTs (Figure S1A), indicating possible anxiety-like behavior in these mice.^24^ A higher cadence (Figure S1B), an increased swing speed as well as stride length (Figures S1D and S1E) also characterized the KO gait.

Mice normally adapt to higher walking speed by increasing the time spent on two paws to the detriment of a three-paws support that is, nevertheless, favored when coping with ataxia or PC degeneration.^28^^,^^29^ WT and KO mice progressively reduced the time they simultaneously placed three paws on the platform during walking to sustain higher walking speeds (Figure 1B). Multiple linear regression analysis showed no significant contribution of the genetic background to the three-paws support adaptation (*p* = 0.34) that was therefore preserved in KO mice. However, the time spent in a three-paws support by KO mice was significantly higher compared to WT (*p* = 0.021) supporting locomotor defects caused by HCAR1 absence. KO walking instability also affected paw placement in these mice, which was characterized by a significantly higher variability of the ipsilateral paw distance while the distance itself was unaffected (Figures 1C and 1D).

As KOs had an increased body weight (Figure S1C) compared to WT mice, we investigated its impact on walking performance. A strong inverse correlation between speed and weight was clearly evident only in KO mice (Figures 1E and 1F), which adapted their pace to their body mass, possibly to overcome locomotor instability (Figures S1F and S1G).

These results support mild gait defects caused by HCAR1 absence that could have a significant impact on motor performance during more demanding tasks. To verify this hypothesis, we used the dataset obtained with the rotarod test (Figures 1G and 1H) by Buscemi et al.^30^. We performed a *de novo* analysis of these data by applying more stringent inclusion/exclusion parameters to highlight possible coordination defects in KO mice (see STAR Methods). Only 31% of KO mice met these new parameters, while most WT mice were included in the analysis (82.9%); the higher propensity of KO mice to be excluded from the analysis was not due to chance (Figure S1H) supporting a compromised performance of these mice on the rotating rod. Indeed, KOs included in the analysis displayed a significant impairment in motor learning; despite performing as well as WTs on the first day of the test, no significant increase in the latency to fall became evident in KO mice by the second or third day of the test. By the second and third day of training, WT mice performed significantly better than KO mice and displayed significant motor learning (Figure 1H). WT and KO mice tested on the rotating rod had similar weight (Figure S1I; it should be noted that the weight was unavailable for 2 of the 34 WT mice tested) and no significant correlation was found between the weight and the latency to fall in both genotypes (Day 1: WT Pearson’s r = −0.027, *p* = 0.88; KO Pearson’s r = 0.25, *p* = 0.51; Day 3: WT Pearson’s r = −0.19, *p* = 0.3; KO Pearson’s r = −0.24, *p* = 0.54).

Our results highlight the role of lactate signaling in motor coordination and show the requirement for HCAR1 signaling for motor learning.

### HCAR1-HCN pathway modulates spontaneous Purkinje cell firing

**Figure 2:**
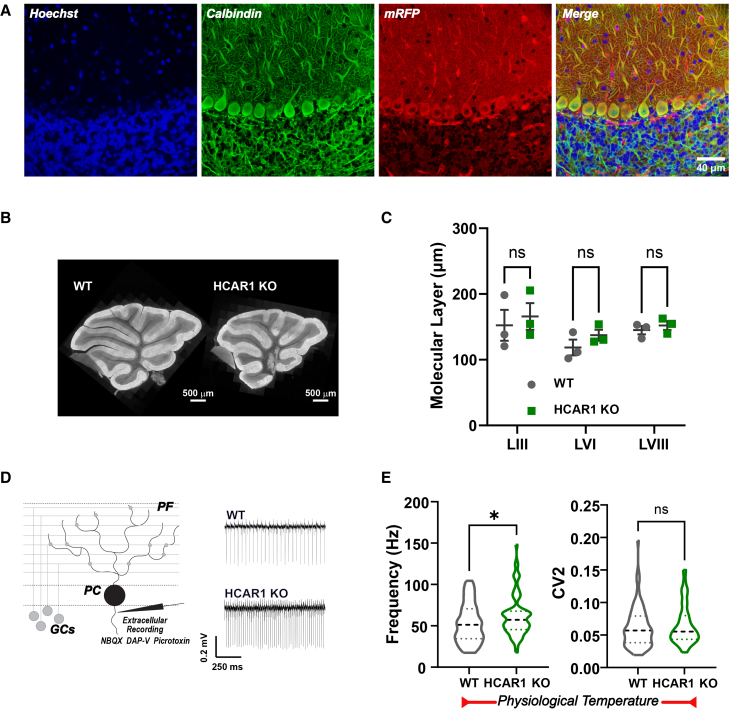
HCAR1 signaling pathway modulates spontaneous Purkinje cells firing (A) Confocal images (maximum projection of 4 z-planes) of cerebellar slice (lobule III) from the GRP81-mRFP murine line stained for mRFP (*mRFP*) and calbindin (*Calbindin*). Nuclei were stained with the fluorescent dye Hoechst (*Hoechst*). Scale bars, 40 μm. (B) Mosaic epifluorescence images of cerebellar slices from WT and HCAR1 KO mice stained for calbindin (*Calbindin*). Scale bars, 500 μm. (C) Scatter plots showing no statistically significant difference in the molecular layer thickness of WT and HCAR1 KO cerebellar slices quantified in lobule III (LIII), VI (LVI) and VIII (LVIII); mean ± SEM, n.s = *p* > 0.05, two-Way ANOVA, WT: *N* = 3, KO; *N* = 3. (D) Schematic representation of spontaneous firing recording from PC (*left*; PC: Purkinje cell, GCs: granule cells, PF: parallel fibers). Representative traces of PC spontaneous activity recorded at near-physiological temperature in WT and KO cerebellar slices are shown on the *right*. E) Violin plots showing a significant increase in PC firing frequency (*left*) in the KOs with no change in the coefficient of variation 2 (*right*, CV2). *Left*: median with the first and third quartile, ∗ = *p* < 0.05, Mann-Whitney test, WT: *N* = 3, *n* = 87; KO: *N* = 5, *n* = 71. *Right*: median with the first and third quartile, n.s = *p* > 0.05, Mann-Whitney test, WT: *N* = 3, *n* = 87; KO: *N* = 5, *n* = 71. Also see Figure S2 and Table S1

**Figure 3:**
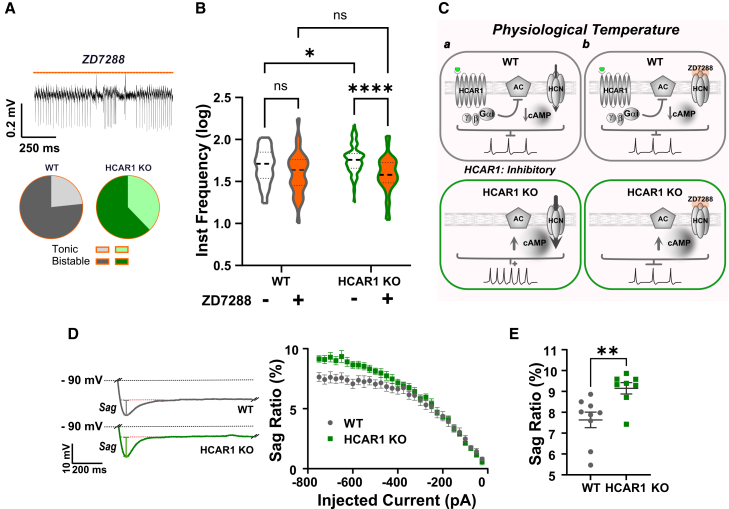
HCAR1-HCN signaling pathway modulates spontaneous Purkinje cells firing (A) Pie chart summarising PC firing behavior in the presence of ZD7288 (*lower*). A representative trace from KO PC showing the alternation of periods of firing and quiescence is shown in the *upper* part. (B) Violin plots summarising the effect of ZD7288 on the instantaneous frequency (logarithmic values) of WT and KO PCs; median with the first and third quartile, n.s = *p* > 0.05, ∗ = *p* < 0.05 and ∗∗∗∗ = *p* < 0.0001, two-Way ANOVA, WT, control: *N* = 3, *n* = 87; WT, ZD7288: *N* = 4, *n* = 30; KO, control: *N* = 5, *n* = 71; KO, ZD7288: *N* = 3, *n* = 45. (C) Schematic summary of the proposed HCAR1 (a) and ZD7288-mediated (b) modulation of spontaneous PC firing at near-physiological temperature (HCAR1: hydroxycarboxylic acid receptors 1, AC: adenylyl cyclase, HCN: cAMP-sensitive hyperpolarization and cyclic nucleotide-gated channels). (D) Representative traces showing the sag of membrane potential (injected current = - 750 pA) in WT and HCAR1 KO PC (*left*) and the scatter plots showing the sag ratio quantified for WT and HCAR1 KO mice at different injected current steps (*right*). *Right*: mean ± SEM, *N* = 3, *n* = 9, KO: *N* = 3, *n* = 8. (E) Scatter plots showing a statistically significant difference in sag ratio characterizing WT and HACR1 KO PCS (Current step = - 750 pA); mean ± SEM, ∗∗ = *p* < 0.01, Mann-Whitney test, WT: *N* = 3, *n* = 9, KO: *N* = 3, *n* = 8. Also see Figure S3 and Table S1.

PCs are spontaneously active^31^^,^^32^^,^^33^ and the alteration of their firing frequency and/or regularity is often associated with motor impairments and ataxias.^34^^,^^35^ HCAR1-mediated tuning of neuronal firing and excitability together with the receptor expression in PCs support a role of HCAR1 as a modulator of their spontaneous firing. We therefore first confirmed HCAR1 expression in PCs by immunostaining of cerebellar slices from GPR81-mRFP mice^27^ used to overcome the lack of specific HCAR1 antibodies^18^; the red fluorescent protein was detected in the cerebellar cortex where it specifically labeled PCs in each lobule as confirmed by co-staining with the specific marker calbindin (Figures 2A and S2A). mRFP reactivity was not uniform throughout the slice, suggesting a possible variable level of expression of the receptor in PCs (Figure S2A). The possible expression of the receptor in the molecular layer (ML) interneurons (stellate and basket cells, MLIs) and granule cells (GCs) was also investigated by immunolabelling of these neurons for the specific markers parvalbumin and NeuN, respectively. Staining for parvalbumin supported the heterogeneous expression of this marker in PCs as previously reported^36^^,^^37^ (Figure S2B). Also, parvalbumin immunoreactivity was identified in MLI somata in the ML (Figures S2B and S2C) and in the basket cell pinceau below PCs, where it colocalized with the red fluorescent protein (Figures S2B and S2D), highlighting HCAR1 expression in MLIs. Furthermore, immunohistochemical analysis also revealed the expression of the receptor in GCs as shown by the colocalization of the red fluorescent protein with the specific marker NeuN (Figure S2E). The overall anatomy of the cerebellum was preserved in KO mice (Figure 2B), and the absence of the receptor had no significant impact on the development of the PC dendritic tree, as shown by the quantification of the ML thickness in the WT and KO mice (Figure 2C). We then proceeded to identify the possible electrophysiological signature of the silenced HCAR1 pathway in KO mice by extracellularly recording spontaneous PC activity in acute cerebellar slices (Figure 2D). The spontaneous activity was recorded from PCs located in the anterior/central (lobules II to VI) and posterior (lobules VIII to X) cerebellar zones similarly sampled in WT and KO cerebellar slices (Figure S2F). At near-physiological temperature, WT PCs were characterized by a significantly lower firing frequency (Figure 2E, *left*) compared to KO PCs, while the firing variability (coefficient of variation 2, CV2) was unaffected (Figure 2E, *right*). Therefore, HCAR1 signaling downmodulates spontaneous PC activity (Figure 3Ca).

HCAR1 is G_i_ protein-coupled, and its activation leads to a decrease in the intracellular cAMP level.^18^ PCs express the cAMP-gated HCN2^38^^,^^39^ and the HCN1 isoform that is characterized by a weak sensitivity to cAMP.^40^ The cAMP-gated isoform HCN4^39^ is likely to be expressed at a very low level, if any.^38^ By decreasing the intracellular cAMP level, HCAR1-mediated signaling could therefore impose a downmodulation of HCN activity, leading to the observed reduced excitability in the WT compared to the KO. We reasoned that blocking HCNs should result in a more pronounced effect on the activity of KO PCs since HCN channels in the mutant should be free from HCAR1-mediated inhibition. We therefore investigated the impact of HCAR1-HCN signaling on spontaneous PC firing by extracellular recording of spontaneous PC activity in the presence of the HCN antagonist ZD7288. Under this experimental condition, PCs revealed their bi-stable nature characterized by the alternation of periods of firing (tonic firing and/or bursting) and quiescence.^41^ Of the recorded cells, 76.7% of WT PCs and 62.2% of KO PCs exhibited bistable behavior (Figure 3A) with no significant influence of the genotype on the firing modality (Fisher’s test, *p* = 0.216). To limit the impact of the firing modality on firing characterization, only periods of tonic firing were further analyzed. The firing variability (CV2) during tonic discharge was significantly increased by ZD7288 bath application both in WT and KO PCs (Figure S3). To account for the ZD7288-introduced temporal variability, we quantified the mean instantaneous frequency (quantified as the inverse of the inter-spike interval at every consecutive spike). The WT and KO no longer showed a statistically significant difference in their firing frequency. As predicted, ZD7288 had a significant impact on spontaneous KO PC firing while WT PCs were mostly unaffected (Figures 3B and 3Cb). Moreover, the sag of the membrane potential observed during the progressive membrane hyperpolarization (Figure 3D) was significantly higher in KO PCs compared to the WT (Figures 3D and 3E), indicating an increased HCN-mediated activity associated with the absence of the receptor.

**Figure 4:**
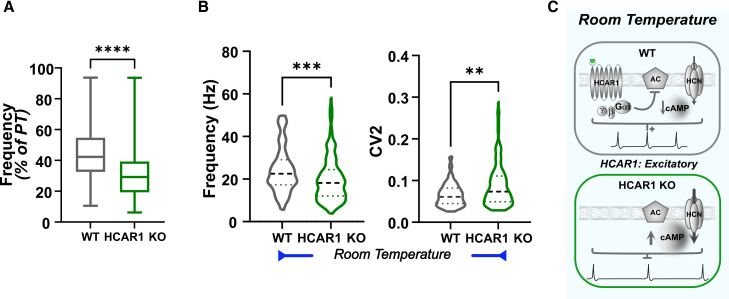
HCAR1 signaling positively modulates spontaneous Purkinje cells firing at room temperature (A) Box and whiskers plot showing the firing frequency obtained from WT and KO PCs recorded at room temperature, normalized to the firing frequency quantified for WT and KO PCs recorded at near-physiological temperature; median, whiskers: minimal and maximal value, ∗∗∗∗ = *p* < 0.0001, Mann-Whitney test, WT: *N* = 3, *n* = 85, KO: *N* = 4, *n* = 153. (B) Violin plots of the firing frequency (*left*) and coefficient of variation 2 (CV2, *right*) of WT and KO PCs recorded at room temperature. *Left*: median with the first and third quartiles, ∗∗∗ = *p* < 0.001, Mann-Whitney test, WT: *N* = 3, *n* = 85, KO: *N* = 4, *n* = 153. *Right*: median with the first and third quartiles, ∗∗ = *p* < 0.01, Mann-Whitney test, WT: *N* = 3, *n* = 85, KO: *N* = 4, *n* = 153. (C) Schematic summary of the proposed HCAR1-mediated modulation of spontaneous PC firing at room temperature (HCAR1: hydroxycarboxylic acid receptors 1, AC: adenylyl cyclase, HCN: cAMP-sensitive hyperpolarization and cyclic nucleotide-gated channels). Also see Figure S4 and Table S1.

To confirm the HCAR1-mediated modulation of spontaneous PC firing via inhibition of HCN channels, we took advantage of HCN heat sensitivity^42^^,^^43^^,^^44^ to indirectly diminish their activity in an attempt to limit the onset of bistable behavior in the recorded neurons. HCN4, HCN2, and HCN1 share common mechanisms to sense heat that increase the channel current density and alter their activation curves.^44^ We therefore recorded spontaneous PC firing at room temperature, an experimental condition expected to reduce the overall contribution of HCN channels to PC activity. Since HCAR1 already provides a partial inhibition of HCN channels in WT PCs, reducing the temperature is expected to have a stronger impact on the spontaneous firing of KO PCs. Lowering the temperature significantly reduced PC firing in both the WT and KO PCs (Figures S4A and S4B). As hypothesized, PC activity displayed a more pronounced sensitivity to the reduced temperature in the absence of the receptor (Figure 4A). Moreover, the firing frequency of WT PCs was higher compared to KO PCs, revealing a net excitatory HCAR1-mediated effect under this experimental condition (Figures 4B, *left* and 4C). A significant increase in firing variability also emerged in KO PCs (Figure 4B, *right*). This temperature-dependent switch of the downstream HCAR1 impact on PC firing revealed its ability to exert a dual modulation over the spontaneous activity of these neurons. HCAR1-mediated excitatory effect also seems to rely on HCNs since it was not observed in the presence of ZD7288.

The HCAR1-mediated excitatory effect also became evident at near-physiological temperature upon the activation of the receptor with the non-metabolized HCAR1 agonist 3Cl-HBA (Figure S4C), indicating the dual modulation provided by the receptor to be an intrinsic property of its signaling pathway also active under physiological conditions.

These results show the neuromodulatory role of lactate signaling in cerebellar physiology, leading to spontaneous PC firing inhibition via HCAR1-mediated tuning of HCN activity. The identification of PCs, GCs, and MLIs as HCAR1-expressing cells highlighted the potential role of HCAR1 in modulating cerebellar circuitry at multiple levels.

### HCAR1-HCN mediated modulation of spontaneous Purkinje cells firing exhibits cell compartment specificity

**Figure 5:**
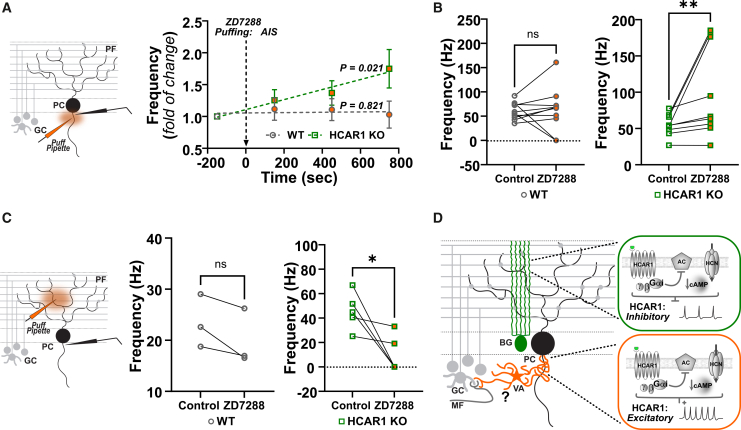
HCAR1-HCN pathway-mediated modulation of spontaneous Purkinje cells firing shows cell compartment specificity (A) (*Right*) Quantification overtime of the firing frequency of WT and KO PCs after ZD7288 local puff at the AIS as drawn on the *left*. Each cell was normalized to its frequency under the control condition (t = −150 s) (dashed lines: linear fitting, correlation analysis: WT Pearson’s r = 0.18 and *p* = 0.821, KO Pearson’s r = 0.98 and *p* = 0.021). Mean ± SEM, WT: *N* = 4, *n* = 9; KO: *N* = 4, *n* = 9. (B) Before-after plots showing ZD7288 effect on WT (*left*) and KO PC (*right*) firing frequency when puffed at the AIS (t = 750 s). *Left*: n.s = *p* > 0.05, paired *t* test, *N* = 4, *n* = 10. *Right*: ∗∗ = *p* < 0.01, Wilcoxon matched-pairs signed rank test, *N* = 4, *n* = 9. (C) Before-after plots showing ZD7288 effect on WT (*center*) and KO PC (*right*) firing frequency when puffed at the dendrites (*left*) (t = 750 s). *Center*: n.s = *p* > 0.05, paired *t* test, *N* = 2, *n* = 3. *Right*: ∗ = *p* < 0.05, paired *t* test, *N* = 3, *n* = 5. (D) Schematic representation of the proposed model for the HCAR1-mediated modulation of spontaneous PC firing (PC: Purkinje cell, GCs: granule cells, PF: parallel fibers; MF: mossy fiber; BG: Bergmann glia, VA: velate astrocytes, HCAR1: hydroxycarboxylic acid receptors 1, AC: adenylyl cyclase, HCN: cAMP-sensitive hyperpolarization and cyclic nucleotide-gated channels). Also see Figure S4 and Table S1.

HCNs contribution to neuronal function is shaped by their expression at a specific neuronal compartment.^45^^,^^46^^,^^47^ In PCs, action potentials originate at the axon initial segment (AIS),^48^^,^^49^^,^^50^ and PC spontaneous activity is modulated by their dendritic tree.^51^^,^^52^ We therefore hypothesized that the HCAR1-mediated bi-directional modulation of PC firing we uncovered was the result of the separate contribution of the HCAR1-HCN pathway at the AIS vs. the dendritic compartment (Figures 5A, *left* and 5C, *left*). Accordingly, we quantified the spontaneous PC firing modulation exerted by ZD7288 when puffed at the putative AIS or at the dendrites. KO PCs responded to HCN blockade at the AIS with a progressive increase in firing frequency (Figures 5A, *right* and 5B, *right*). Puffing of HEPES-based aCSF at the AIS also caused a mild significant increase in firing frequency (Figure S5B) that was, nevertheless, significantly lower compared to the ZD7288-mediated effect (Figure S5C) on KO PCs.

The ZD7288-mediated increase in firing frequency ended in bistable behavior in 44.4% of the neurons. Bistable behavior was also present in 40% of WT PCs but the antagonist was unable to induce a significant change in action potential discharge during tonic firing (Figures 5A, *right* and 5B, *left*). Similar results were obtained when the instantaneous frequency was quantified (WT: paired *t* test, *p* = 0.89; HCAR1 KO: Wilcoxon matched-pairs signed rank test, *p* = 0.0078).

ZD7288 puffing at the dendrites caused an overall mild depression of KO PC firing while a small increase was observed in WT PCs (Figures S5D–S5F). Within each group, however, only a fraction of the recorded neurons showed a decrease in firing frequency in response to the antagonist puff (WT: 37.5%; KO: 41.7%) while a net increase was observed in the remaining cells. In control experiments, puffing of HEPES-based aCSF at PC dendrites also raised the firing frequency both in WT (Figure S5G) and KO PCs (Figure S5H), indicating that the observed ZD7288-mediated excitatory effect was likely nonspecific under our experimental condition. We therefore quantified the impact of blocking HCN channels on firing behavior only in neurons inhibited by ZD7288. In these neurons, a significant decrease in the firing frequency was evident only in KO PCs. (Figure 5C). Similar results were obtained when the instantaneous frequency was quantified (WT: paired *t* test, *p* = 0.114; HCAR1 KO: paired *t* test, *p* = 0.045).

Our results support a possible cell compartment-specific HCN-mediated modulation of spontaneous PC firing in the KOs. HCNs located at the AIS negatively regulate action potential discharge, whereas PC firing is facilitated by HCN channels expressed at the dendrites.

## Discussion

In this paper, we characterized the unexplored role of the lactate receptor HCAR1 in motor behavior and cerebellar physiology. The knockout of the receptor in mice led to gait instability and motor learning impairment compatible with a cerebellar origin of these defects. HCAR1 expression was identified in PCs, GCs, and MLIs in the GPR81-mRFP reporter mouse, highlighting the potential ability of the receptor to influence the input and output stages of the cerebellar cortex. The immunoreactivity of mRFP-positive cells was not uniform, suggesting a variable level of HCAR1 expression. However, in the absence of a reliable antibody against the HCAR1 protein, a detailed characterization of the receptor expression in WT mice is precluded, and no definitive conclusion can be drawn at this time.

Motor defects were associated with the enhancement of spontaneous PC firing in HCAR1 KO mice, highlighting HCAR1-mediated modulation of PC activity and further supporting the expression of the receptor in these neurons. Since the spontaneous firing of these neurons was recorded in the majority of the cortical lobules, with WT and HCAR1 KO PCs similarly sampled across the cerebellar zones, any variability in receptor expression is expected to minimally contribute to the observed inhibitory effect mediated by HCAR1 under these experimental conditions.

Pharmacology experiments, whole-cell electrophysiological recordings, and the enhanced heat sensitivity displayed by KO PCs supported HCN channels as the downstream targets of the receptor. The HCAR1-HCN signaling pathway had opposite downstream effects on PC firing, consistent with a differential role of HCNs at the AIS and dendrites.

Spontaneous PC firing is intrinsically determined by diverse types of ionic conductance active at the axo-somatic and dendritic level. HCN channels have been shown to have negligible effects on spontaneous PC firing rate^40^^,^^41^ but to control membrane potential bistability of these neurons by stabilizing the membrane voltage within a range optimal to sodium channels function.^41^ In agreement, we showed a limited ZD7288-mediated effect on spontaneous WT PC tonic firing frequency and the bistable behavior of these neurons unleashed by HCNs blockade. However, silencing HCAR1 uncovered PC firing frequency sensitivity to ZD7288 and the cell compartment-specific modulation provided by HCNs, indicating that the antagonist effect was likely occluded in WT PCs by HCAR1 activation by ambient lactate.

We exploited the absence of HCAR1-mediated inhibition of HCN channels in KO mice to further investigate HCN-mediated modulation of spontaneous PC firing. Local application of ZD7288 at the AIS stimulated PC firing that was nevertheless inhibited by the antagonist when puffed at the dendrites.

Action potentials passively back-propagate into the dendrites^49^^,^^50^ because of limited sodium channel expression at this location. Nevertheless, PC dendrites generate plateau potential and calcium spikes which can propagate to the soma.^52^^,^^53^ In tonically firing PCs, dendrites provide a net inward current to the soma, modulating excitability in these neurons.^51^ However, blocking calcium channels at the dendrites has negligible effects on PC firing rate,^51^ suggesting that other ion channels support the dendrite-mediated depolarizing drive to the soma during tonic firing. When puffed at KO PC dendrites, ZD7288 silenced 60% of the recorded neurons and decreased the firing frequency in the remaining cells. Our results therefore indicate that HCN at the dendrites might contribute to the inward current that sustains PC excitability.

At the AIS, ZD7288 increased tonic PC firing, revealing HCN ability to dampen PC action potential discharge in contradiction with their established role in pace-making activity.^54^^,^^55^^,^^56^^,^^57^^,^^58^ However, ZD7288-mediated PC membrane voltage hyperpolarization^41^^,^^59^ could favor low-threshold voltage-gated calcium channels (T-type) de-inactivation, thereby increasing their contribution during the inter-spike interval.^32^ Enhancing T-type calcium channel activity will favor membrane depolarization and therefore sodium channel opening, causing an overall increase in firing frequency upon HCN blockade or via HCAR1-mediated signaling.

We propose the following model to explain the complex modulation of spontaneous PC firing mediated by HCAR1-HCN signaling (Figure 5D). The overall effect of HCAR1 activation in PCs is the downmodulation of HCNs and their contribution to PC excitability. HCAR1-G_i_ signaling^18^ is expected to decrease the intracellular cAMP level and negatively affect the cAMP-gated HCN isoforms by shifting their activation curve toward more hyperpolarized potentials.^60^ HCN2 higher sensitivity to this second messenger predicts a more pronounced effect of HCAR1 signaling on this subunit over HCN1. Hoverer, HCNs can form heterotetramers^61^^,^^62^^,^^63^^,^^64^; the assembly of HCN2 with HCN1 subunits into heterometric complexes^61^ may therefore enable HCAR1 to also exert a more powerful control over the HCN1 subunit. The role of HCN4 cannot be excluded at this time, but the very low expression of this isoform suggests a negligible contribution of this channel to PC excitability. The HCAR1-HCN pathway modulates PC excitability in a cellular compartment-dependent way. At the dendrites, HCAR1-mediated downmodulation of HCN channels limits the excitatory effect mediated by this compartment on the soma, while at the AIS it sustains repetitive firing. These two mechanisms coexist as highlighted by the puffing experiments; however, at near-physiological temperature, the dendritic HCAR1-HCN pathway seems to predominate over the one at the AIS, as indicated by the lower firing frequency displayed by WT PCs when compared to KO PCs. Nevertheless, the increase in PC firing frequency caused by the agonist-mediated activation of the receptor raises the interesting possibility for the HCAR1-HCN pathway at the AIS to be more effectively engaged during times of intense neuronal activity when extracellular lactate concentration rises. Neuronal activity stimulates lactate release from astrocytes, causing a rapid increase in the extracellular lactate concentration. Different classes of astrocytes occupy distinct regions of the cerebellar cortex and target specific neuronal populations.^65^ In the ML, BG enwrap excitatory synapses at PC dendrites, and they are recruited by sensorimotor information and error/predicting signals reaching the cerebellum via the mossy fiber (MF)- GC afferent system and CF, respectively.^66^^,^^67^^,^^68^^,^^69^^,^^70^^,^^71^^,^^72^ In the granule cell layer (GCL), velate astrocytes (VA) modulate MF to GC transmission^73^ at the MF glomeruli,^74^ and they are therefore engaged by sensorimotor information at the input stage of the cerebellar cortex. Astrocytic processes are found beneath PC soma where they wrap the axon^75^; their location within the GCL together with their positivity to the sulfate proteoglycan brevican^74^ suggest their origin to be from VA. This raises the possibility for PCs to be contacted by BG and VA, and for lactate to be released from these two classes of astrocytes in order to engage HCAR1 signaling at different cellular compartments. We therefore propose that HCAR1 signaling endows PCs with the ability to scale up or down their excitability in response to the processing of sensorimotor information performed at different cortical levels and relayed through the recruitment of BG or VA (Figure 5D).

The heterogeneity of GPCR signaling cascades and their targets predicts that alternative HCAR1 downstream pathways are likely to work in parallel with the HCAR1-HCN axes identified to shape PC activity and modulate cerebellar physiology.

The activation of G protein-coupled inwardly rectifying potassium (GIRK) channels has been described as a possible HCAR1 target in subicular neurons.^76^ GIRK channels are expressed in PCs, and their activation reduces PC excitability, prevents long-term depression (LTD), and enhances long-term potentiation at GC to PC synapses.^77^ The HCAR1-GIRK pathway could therefore confer to lactate signaling an alternative route to modulate PC firing and influence cerebellar-mediated motor learning by regulating synaptic plasticity.

HCAR1-mediated inhibition of PKA could negatively impact PC activity-dependent intrinsic plasticity that is induced by downmodulation of SK channels, and it is dependent on the activation of this kinase.^78^ Furthermore, HCAR1 expression in GCs could interfere with the induction of a presynaptic PKA-dependent form of LTP at GC to PC synapses.^79^^,^^80^^,^^81^^,^^82^ Moreover, HCAR1 expression in MLIs supports a role of lactate signaling in modulating the activity of these interneurons, which in turn will allow the receptor to participate in the fine-tuning of PCs activity^83^^,^^84^ and in the modulation of PC calcium dynamics^85^^,^^86^^,^^87^ long-term plasticity at GC to PC synapses^86^^,^^87^ and cerebellar-mediated motor learning.^87^

The complex modulation of PC activity provided by HCAR1 together with its expression at multiple levels of the cerebellar cortical microcircuit highlights a central role of lactate signaling in cerebellar physiology that we only start to comprehend. The gait instability and motor learning defects identified in KO mice support the significant role of HCAR1 in cerebellar function, motor learning, and cerebellar-mediated diseases.

### Limitations of the study

The gait instability and motor-learning defects identified in HCAR1 KO mice support the cerebellum as the major player in the establishment of these altered phenotypes. Because the mouse model used in this study is a full knockout, the involvement of other brain regions cannot be excluded at this time. A more targeted approach, specifically silencing HCAR1-mediated signaling only in the cerebellum, would be required to confirm the pure cerebellar origin of the defects highlighted in this study.

The absence of HCAR1-specific antibodies prevented a detailed *in situ* characterization and quantification of the receptor protein expression in the cerebellar cortex of the WT mice used in this study. While the identified HCAR1-mediated modulation of the spontaneous PC firing strongly supports the expression of the receptor in these neurons, its possible role in MLIs and GCs physiology remains to be addressed.

Because only male mice were used in this study, the influence of sex on the described phenotypes could not be addressed.

## Resource availability

### Lead contact

Requests for further information, resources or material should be directed to the lead contact Prof. Jean-Yves Chatton (jean-yves.chatton@unil.ch).

### Materials availability

This study did not generate new unique reagents.

### Data and code availability

• Data have been deposited at https://doi.org/10.5281/zenodo.20341820 and are publicly available as of the date of publication.
• This paper does not report original code.
• Any additional information required to reanalyze the data reported in this paper is available from the lead contact upon request.

## Acknowledgments

We thank Dr Leonardo Restivo (NeuroBau, University of Lausanne) for his technical assistance and help with the behavioral tests of this study. We thank Dr Lior Bikovski (Behavioral Testing Core Facility, Tel Aviv University) for his valuable input for the analysis of the catwalk experiments. We thank Dr Frédéric Schütz (Biostatistics Platform, University of Lausanne) for his help with the statistical analysis. We thank Dr Nadia Rosenberg, Fanny Thevenaz, Claudia Casto and Christiane Devenoges for their technical assistance. We would like to thank Prof. Stefan Offermanns at the Max Planck Institute for Heart and Lung Research in Germany for designing the HCAR1 KO and GRP81-mRFP mouse lines and sharing them with us.

We would like to thank the 10.13039/501100001711Swiss National Science Foundation for the financial support to our work (grant nos. 31003A_179399 and 310030_212432 and 31003A_179399 to J.Y.C. and 310030_212233/1 to L.H.).

F.B. thanks Dr Maxime Alessandri for having made this journey easier and fun.

## Author contributions

F.B. conceptualized the study, performed electrophysiological recording, immunohistochemistry, morphological analysis and the Catwalk test, collected and analyzed the data, wrote the manuscript; L.B. conceptualized and performed rotarod experiments; N.G. performed electrophysiological recordings; L.H. conceptualized rotarod experiments; J.Y.C. conceptualized the study and wrote the manuscript.

## Declaration of interests

Authors declare no competing interests.

## STAR★Methods

### Key resources table

**Table:** 

REAGENT or RESOURCE	SOURCE	IDENTIFIER
**Antibodies**		

Rabbit polyclonal anti-Calbindin	Swant	Cat# cb38a; RRID:AB_3107026
Rabbit polyclonal anti-Parvalbumin	Swant	Cat# PV-28; RRID:AB_2315235
Mouse monoclonal Anti-NeuN	Sigma-Aldrich	Cat# MAB377; RRID:AB_2298772
Goat polyclonal anti-TdTomato	MyBiosource	Cat# MBS448092; RRID:AB_2827808
Donkey polyclonal secondary anti-Mouse Alexa Fluor 488	Thermo Fisher Scientific	Cat# A32766; RRID:AB_2762823
Donkey polyclonal secondary anti-Rabbit Alexa Fluor 488	Thermo Fisher Scientific	Cat# A-21206; RRID:AB_2535792
Donkey polyclonal secondary anti-Goat Alexa Fluor 594	Thermo Fisher Scientific	Cat# A-11058; RRID:AB_2534105

**Chemicals, peptides, and recombinant proteins**		

Adenosine 5′-triphosphate disodium salt hydrate	Sigma-Aldrich	Cat# A2383
Calcium chloride	Sigma-Aldrich	Cat# C5670
Ethylene glycol-bis(2-aminoethylether)-*N*,*N*,*N′*,*N′*-tetraacetic acid (EGTA)	Sigma-Aldrich	Cat# CE4378
Donkey serum	Sigma-Aldrich	Cat# D9963
FluorSave^TM^ Reagent	Millipore	Cat# 345789
D-Glucose	Sigma-Aldrich	Cat# RDD016
Guanosine 5′-triphosphate sodium salt hydrate	Sigma-Aldrich	Cat# G8877
HEPES	Sigma-Aldrich	Cat# H3375
Hoechst 33258	Thermo Fisher Scientific	Cat# H1398
Paraformaldehyde	Electron Microscopy Sciences	Cat# 15710-S
Phosphate Buffered Saline	biowest	*X*0515
Phosphocreatine disodium salt hydrate	Sigma-Aldrich	Cat# P7936
Potassium chloride	Sigma-Aldrich	Cat# 60130
Potassium D-gluconate	Sigma-Aldrich	Cat# G4500
Magnesium chloride hexahydrate	Sigma-Aldrich	Cat# M2670
Sodium chloride	VWR BDH Chemicals	Cat# 27800.291
Sodium bicarbonate	Sigma-Aldrich	Cat# S5761
Sodium phosphate monobasic dihydrate	Sigma-Aldrich	Cat# 71505
Sucrose	Fisher Chemical	Cat# S/8600/60
Triton X-100	Fluka	Cat# 93420
3-chloro-5-hydroxybenzoic Acid (3Cl-HBA)	Selleckchem	Cat# S5400
D-AP5	Tocris	Cat# 0106
NBQX	Tocris	Cat# 0373
Picrotoxin	Tocris/Sigma-Aldrich	Cat# 1128/P1475
ZD7288	Tocris	Cat# 1000

**Deposited data**		

Original data	This paper	https://doi.org/10.5281/zenodo.20341820

**Experimental models: Organisms/strains**		

Mouse: C57Bl6/N	In house colony	NA
Mouse: HCAR1 KO	Ahmed et al.^25^	NA
Mouse: GPR81-mRFP	Ahmed et al.^25^	NA

**Software and algorithms**		

pClamp	Molecular Device	RRID:SCR_011323
GraphPad Prism	Dotmatics	RRID:SCR_002798
ImageJ	Schindelin et al.^73^	RRID:SCR_003070

### Experimental model and study participant details

#### Mice

Experiments were performed in agreement with Swiss National Institutional Guidelines on animal experimentation and were approved by the Vaud Cantonal Veterinary Office (authorizations VD1288 and VD2017.6A).

Homozygous HCAR1 KO (C57BL6/N background, homozygous breeding), C57BL6/N and GPR81-mRFP male mice were used for experiments and randomly assigned to experimental groups. For each experimental procedure, the mice age is indicated in the corresponding paragraph in the “method details” section.

Mice were housed with free access to food and water and a 12 hours light/dark cycle.

Experiments and analysis were performed by researchers not blind to the genetic background of the mice.

### Method details

#### Acute cerebellar slices

Mice within P24-P75 days of age have been used for cerebellar acute slice preparation and electrophysiological recordings. Mice were anesthetized by isoflurane exposure, decapitated and the cerebellum rapidly dissected in ice-cold oxygenated (constantly bubbled with a 95% O_2_/5% CO_2_ mix) dissection medium containing (in mM): NaCl 120, KCl 3, NaH_2_PO_4_ 1.25, NaHCO_3_ 26, CaCl_2_ 0.5, MgCl_2_ 4 and glucose 20. Sagittal 290 μm thick slices were prepared using a Leica Vibratome 1000S in ice-cold oxygenated slicing solution containing (in mM): sucrose 240, KCl 3, NaH_2_PO_4_ 1.25, NaHCO_3_ 26, CaCl_2_ 0.5, MgCl_2_ 4 and glucose 20. At the end of the slicing procedure, cerebellar slices were transferred to a recovery chamber containing 150 ml of slicing solution (constantly bubbled with a 95% O_2_-5% CO_2_ mix) and held at 34°C. We adopted the sodium spike recovery method to improve slice quality.^88^ Briefly, the concentration of sodium inside the recovery chamber was slowly raised by the repetitive sequential addition of increasing volumes of a NaCl 2M solution (dissolved in slicing solution; 250 μl/ twice, 500 μl, 1 ml and 2 ml) delivered at fixed time intervals determined by the age of the animal (first addition before incubating the slices and then every two minutes for mice younger than 1month and every five minutes for mice older than 1 month). Slices were then transferred to a holding chamber filled with 95% O_2_-5% CO_2_ bubbled aCSF containing (in mM): NaCl 120, KCl 3, NaH_2_PO_4_ 1.25, NaHCO_3_ 26, CaCl_2_ 2, MgCl_2_ 1 and glucose 20. The holding chamber was maintained at room temperature (25°C) for the rest of the day and slices were allowed to recover for at least 30 minutes before starting the recording.

#### Electrophysiology

##### Spontaneous firing recording

Electrophysiological recordings were performed with a MultiClamp 700B amplifier in current-clamp mode (Molecular Devices) and data acquired using the Clampex software (Molecular Devices).

For electrophysiological recording, the cerebellar slice was placed in the recording chamber and constantly perfused with 95% O_2_-5% CO_2_ bubbled aCSF. Picrotoxin 100 μM, D-APV 50 μM and NBQX 5 μM were added to the aCSF to block GABAergic and glutamatergic transmission.

The recording of spontaneous PC activity was performed at near-physiological temperature (32-34°C) or room temperature (25°C) as indicated in the text. Spontaneous PC firing was extracellularly recorded by placing a recording borosilicate glass pipette in the granule cell layer (GCL) in close proximity to the PC soma. The recording pipette had a resistance of 1.2-4.1 MΩ when filled with NaCl 0.5 M. Recordings were filtered at 2 kHz and digitized at 10 kHz via a Digidata 1440A (Molecular Devices). PC spontaneous activity was recorded for 150 seconds and only cells firing tonically for the entire duration of the recording were included in the analysis. Spontaneous activity was recorded from PCs located in lobules II to VI and lobules VIII to X.

HCN contribution to spontaneous PC activity was investigated at near-physiological temperature by bath application of the specific antagonist ZD7288 at a final concentration of 10 μM (stock solution was prepared by dissolving ZD7288 in water). Tonically firing PCs and PCs exhibiting a bistable behavior were included in the analysis but, for the latter group, firing frequency and variability were quantified only during randomly chosen periods of tonic firing.

##### ZD7288 puffing experiments

Puffing experiments were performed using a Picospritzer II (General Valve Corporation). Control experiments were performed by the sole puffing of the HEPES-based aCSF solution containing in mM: NaCl 120, KCl 3, NaH_2_PO4 1.25, HEPES 10, CaCl_2_ 2, MgCl_2_ 1 and glucose 20. ZD7288 (10 μM in HEPES-based aCSF) was delivered via a borosilicate pipette either to the putative AIS or to the dendrite of the recorded PC. The baseline spontaneous activity was recorded for 150 sec before starting the puffing. At the end of the puffing protocol, the spontaneous activity was followed overtime and the firing frequency quantified in consecutive 150 sec long intervals. In bistable neurons, the firing frequency was quantified only during randomly chosen periods of tonic firing.

Spontaneous activity was recorded from PCs located in lobules IV to VI.

##### AIS puffing

To place the puffing pipette, we used the recording electrode as guidance. Sodium channels are found with high density at the AIS therefore extracellular recorded action potentials at this location should be characterized by higher amplitude compared to other locations. We therefore initially placed the recording pipetted in the GCL beneath and close to the soma of the PC and the position was adjusted to maximize the recorded spikes amplitude. The puffing pipette was then placed in proximity to the recorded cell and facing the recording pipette. The puffing protocol consisted of 5 puffs delivered at 1 minute intervals for 10 times. Each puff was 500 ms long and the inter-puff interval was set to 50 ms.

##### Dendrite puffing

The puffing pipette was initially placed in the middle of the molecular layer. Halfway through the puffing protocol, the puffing pipette was moved toward the distal end of the molecular layer (ML).

The puffing protocol consisted of 5 puffs delivered at 1 minute interval for10 times. Each puff was 800 ms long and the inter-puff interval was set to 50 ms.

##### 3Cl-HBA experiments

Spontaneous PC activity was recorded for 150 seconds in lobule VI and only cells firing tonically for the entire duration of the recording were included in the analysis. The spontaneous firing was quantified under control condition and following the bath application of the non-metabolized HCAR1 agonist 3Cl-HBA to a final concentration of 40 μm.

##### Whole-cell recording

Whole cell recording from PCs located in lobule IV to VII were performed in the current-clamp mode with borosilicate pipettes with a resistance of 4-8 MΩ when filled with the intracellular recording solution containing in mM: K Gluconate 130, KCl 10 mM, MgCl_2_ 2, HEPES 10, Na_2_ATP 4, NaGTP 0.4, EGTA 0.1 and Phosphocreatine 10 (pH adjusted to 7.3). Recordings were performed at near-physiological temperature in presence of picrotoxin 100 μM, NBQX 5 μM and D-APV 50 μM. PC were held near – 70 mV and the membrane potential progressively hyperpolarized by current injections in -25 pA increment (1-3 seconds long step). The sag of the membrane potential was quantified as the difference between the maximum membrane voltage reached upon hyperpolarization (peak_V_) and the membrane voltage reached at the end of the current step. The sag ratio was quantified as: sag ratio (%) = (sag/peak_V_)∗100. Recordings were filtered at 2kHz and digitized at 10 kHz.

#### Motor behavior analysis

##### Catwalk test

Mice within P33-P36 days of age were used for the Catwalk test. WT and KO mice were gently handled daily by the researcher over a period of 3 days prior to the start of the behavioral test to familiarize the mice with the researcher and to reduce stress. The Catwalk test (CatWalk XT, Noldus) consisted of 3 days of habituation of the mouse to the walkway and 2 days of testing. During the habituation period, mice were individually placed on the walkway and allowed to explore it for 3 minutes before being returned to the home cage. On the two days of testing, 3-6 runs/mouse were acquired per day and used for offline quantification and analysis. Parameters quantified for each run over the two days of testing were averaged together and statistical analysis performed.

##### Rotarod test

To comply with the 3Rs rule for animal experimentation, we did not run *de-novo* rotarod behavioral testing on WT and KO mice, but we exploited the availability of our previously collected data set to evaluate the role of HCAR1 during ischemic stroke. The results presented in this work originate from the original data collected and previously published by Buscemi et al. 2022.^30^ A *de-novo* analysis was performed to identify motor impairments associated with the absence of the HCAR1 receptor. Specifically, only data obtained from WT and KO mice testing before transient middle cerebral artery occlusion (MCAO) were used in our study. Mice subjected to MCAO were expected to cope with the ischemic lesion during the rotarod test by grabbing the rod (instead of running on it) therefore trials during which mice grabbed the rotating rod were included in the original analysis. With the aim to uncover motor coordination defects in KO mice and to quantify the latency to fall exclusively describing motor performance originating from active walking, we excluded these trials from our analysis. Specifically, only trials successfully completed by the mouse or ended because of falling from the rod were included in the analysis while all trials during which the tested mouse stopped walking but did not fall by grabbing the rod were excluded; to be included in the experimental group, mice required at least one valid trial/day.

For the rotarod test, 8-12 weeks old male mice were used and motor coordination was evaluated by placing the mice on an accelerating rotating rod (Ugo Basile). From a starting velocity of 4 rpm, acceleration was set to reach 40 rpm in 216 seconds and a constant rotation speed of 40 rpm was maintained thereafter until the end of the trial at 300 seconds from the start. Mice underwent 3 trials/day for a total of 3 days of testing. For each day of testing, the mean latency to fall was quantified by averaging the trials compliant with the new inclusion/exclusion parameters.

#### Immunostaining and microscopy

One GRP81-mRFP male mouse (P40) was intracardially perfused with PFA 4% after terminal anesthesia with pentobarbital (150 mg/kg) and the cerebellum dissected. Following overnight post fixation in PFA 4%, sagittal cerebellar slices (100 μM) were prepared using a Leica 1000S vibratome and used for immunostaining. Slices were incubated for 3 hours at room temperature in blocking solution containing: donkey serum 10% and Triton X-100 0.5% (in PBS). The rabbit anti-Calbindin (1:1000 ), the rabbit anti-Parvalbumin (1:1000), the mouse anti-NeuN (1:200) and the goat anti-tdTomato (1:50 ) were used as primary antibodies and diluted in in blocking solution. Slices were initially incubated overnight at 4°C with the anti-tdTomato primary antibody; the primary antibody against Calbindin, Parvalbumin or NeuN was then added and slices were incubated with the two primary antibodies overnight at 4°C. Primary antibodies were removed by three consecutive washes in PBS. The Alexa Fluor 488-conjugated donkey anti-rabbit , the Alexa Fluor 488-conjugated donkey anti-mouse and the Alexa Fluor 594- conjugated anti-goat were used as secondary antibodies at a dilution of 1:500 in blocking solution. Slices were incubated with the two secondary antibodies at room temperature for 3 hours. At the end of the incubation, an initial wash in PBS was followed by nuclei staining with Hoechst (1:250 in PBS) for 15 minutes. Slices were then washed 3 times with PBS and mounted on glass microscope slides with the mounting medium FluorSave.

Z-stack series of 1024x1024 pixels images were acquired with the Leica Stellaris 8 confocal and a 63x oil immersion objective. The mosaic image was acquired with a 20x objective.

For representative reasons only, the images contrast was equally adjusted in each channel by equalization of the image histogram by a 0.02 %-0.2 % factor with the ImageJ software.^89^

Epifluorescence mosaic images were acquired with the Leica DMI8 epifluorescence microscope and a 40x oil immersion objective.

#### Morphological analysis

The molecular layer thickness was quantified in sagittal cerebellar slices from three WT and HCAR1 KO mice that previously underwent to Catwalk behavioral testing. Mice were intracardially perfused with PFA 4% after terminal anesthesia with pentobarbital (150 mg/kg) and the cerebellum dissected. Following overnight post fixation in PFA 4%, sagittal cerebellar slices (50 μM) were prepared using a Leica 1000S vibratome and used for calbindin immunostaining as previously described. Quantification of the molecular layer thickness was performed using the ImageJ software. The height of the molecular layer was measured in lobule III, VI and VIII by measuring the distance between the upper limit of the PC layer and the slice surface perpendicularly to the surface. Measurements were taken at six separate locations within each lobule in two slices/mouse analyzed and averaged together.

### Quantification and statistical analysis

Electrophysiological data were acquired with the Clampex software and analyzed offline. Raw traces were adjusted for baseline drifting and action potentials discharge was quantified with the Threshold method of the Clampfit software; the threshold was visually set by the researcher The coefficient of variation 2 (CV2) was calculated with the mathematical formula: CV2 = 2ǀISI_n+1_ - ISI_n_ǀ/(ISI_n+1_+ ISI_n_).

Statistical analysis was performed with the GraphPad Prism software. Normal distribution of the data was tested with the D’Agostino-Pearson test or Shapiro-Wilk test and QQ plots and parametric or non-parametric tests were applied accordingly as indicated in the Table S1.

Two-way ANOVA test was applied to data not following the Gaussian distribution but with a lognormal distribution (D’Agostino and Pearson test) after their conversion into logarithmic values.

Data are presented as follows: bar graph: mean ± SEM; scatter plot: mean ± SEM and individual values; box and whiskers plot: median, whiskers: minimal and maximal value; violin plot: median with the first and third quartile. Significance was set at *p* < 0.05 and shown in the figures as follows: n.s = *p* > 0.05; ∗ = *p* < 0.05; ∗∗ = *p* < 0.01, ∗∗∗ = *p* < 0.001; ∗∗∗∗ = *p* < 0.0001.

N indicates the number of mice and n indicates the number of cells. N and *n* values for each figure can be found in Table S1.
