# Supplementary material for: HCAR1-mediated lactate signaling modulates motor behavior and regulates spontaneous firing in Purkinje cells
Source: iScience. 2026 Aug 3;29(8):116930. doi: 10.1016/j.isci.2026.116930 (PMC13470098; doi:10.1016/j.isci.2026.116930)
Supplement: Supplementary file 1 [file mmc1.pdf]

iScience, Volume *29*

Supplemental information

HCAR1-mediated lactate signaling modulates motor behavior and regulates spontaneous firing in Purkinje cells

Francesca Binda, Lara Buscemi, Nicolas Gurtner, Lorenz Hirt, and Jean-Yves Chatton

Supplementary Figures

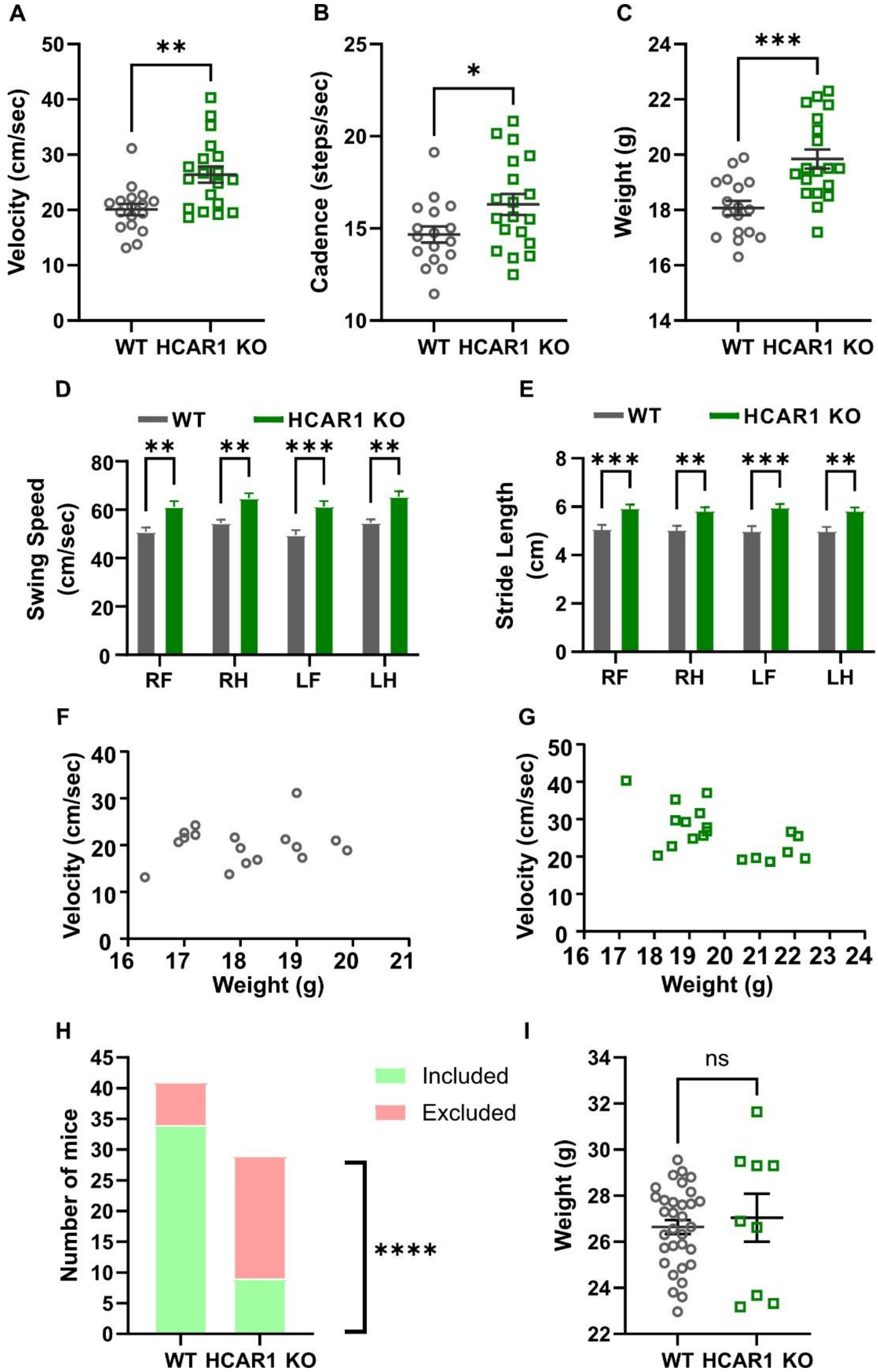

Figure S1: The lactate receptor HCAR1 modulates motor behaviors in mice

A: Scatter plots showing the increase in walking velocity in KO mice compared to WTs. Mean ± SEM, ** = P < 0.01, Unpaired t-test, WT: N = 17, KO: N = 19.

B: Scatter plots showing the increased cadence characterizing KO mice when compared to WTs. Mean ± SEM, * = P < 0.05, Unpaired t-test, WT: N = 17, KO: N = 19.

C: Scatter plots showing the increase in body weight characterizing the KO compared to the WT. Mean ± SEM, *** = P < 0.001, Unpaired t-test, WT: N = 17, KO: N = 19.

D: Bar graphs showing the swing speed quantified for each paw in WT and KO mice. The swing speed of each paw is higher in the KO compared to the WT (paws: right front, RF; right hind, RH; left front, LF; left hind, LH). Mean ± SEM, ** = P < 0.01, *** = P < 0.001, Two-way ANOVA, WT: N = 17, KO: N = 19.

E: Bar graphs showing the stride length quantified for each paw in WT and KO mice. The stride length of each paw is higher in the KO compared to the WT (paws: right front, RF; right hind, RH; left front, LF; left hind, LH). Mean ± SEM, ** = P < 0.01, *** = P < 0.001, Two-way ANOVA, WT: N = 17, KO: N = 19.

F: Scatter plots showing the absence of velocity-weight correlation in WT mice (symbols: single mouse).

G: Scatter plots showing the velocity-weight correlation in KO mice (symbols: single mouse).

H: Bar graphs showing the number of mice tested with the rotarod (dataset from Buscemi et al. 2022 [1]) and included/excluded in the experimental groups because of the new inclusion criteria applied to highlight motor coordination impairment. **** = P < 0.0001, Fisher's test, WT: N = 41, KO: N = 29. Also see Table S1.

I: Scatter plot showing the weight of WT and HCAR1 meeting the new inclusion criteria and tested on the rotating rot (symbols: single mouse). n.s = P > 0.05; Unpaired t-test, WT: N = 32, KO: N = 9.

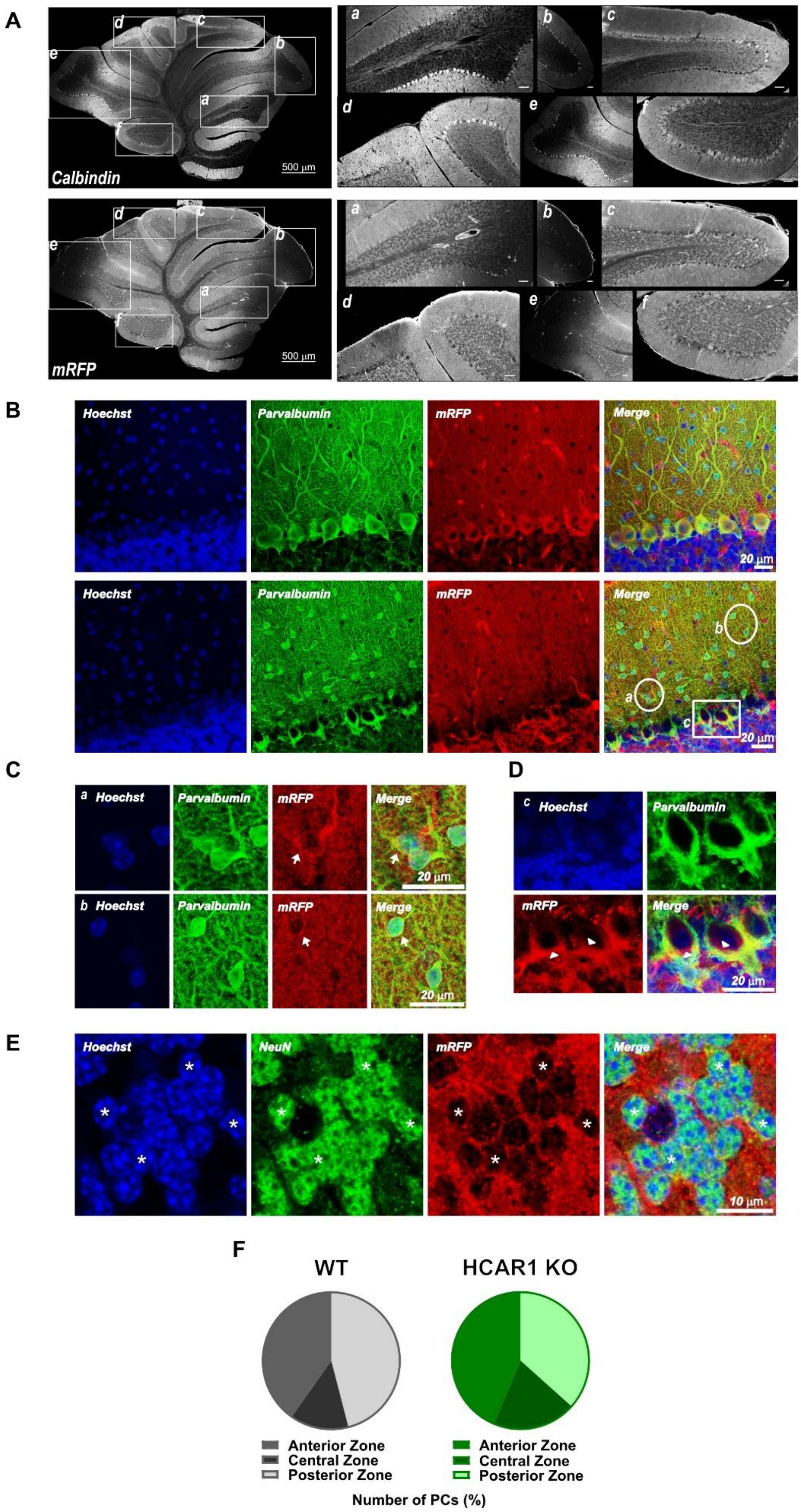

Figure S2: HCAR1 expression in the cerebellum

A: Confocal mosaic images (single plain) of one cerebellar slice from the GRP81-mRFP murine line stained for mRFP (*mRFP*) and calbindin (*Calbindin*) are shown in the left panel (scale bars: 500 µm). Enlargements of the area highlighted in white (*a-e*) are shown in the right panel (scale bars: 50 µm).

B: *Upper panels:* confocal images (maximum projection of 7 z-planes) of cerebellar slice (lobule III) from the GRP81-mRFP murine line stained for mRFP (*mRFP*) and parvalbumin (*Parvalbumin*). Nuclei were stained with the fluorescent dye Hoechst (*Hoechst*). Scale bars: 20 µm.

Lower panels: confocal images (maximum projection of 10 z-planes) of cerebellar slice (lobule VI) from the GRP81-mRFP murine line stained for mRFP (*mRFP*) and parvalbumin (*Parvalbumin*). Nuclei were stained with the fluorescent dye Hoechst (*Hoechst*). Scale bars: 20 µm.

C: Enlargement of area *a and b* of panel B. Arrows indicate mRFP staining in MLIs. Scale bars: 20 µm.

D: Enlargement of area *c* of panel B. Arrowheads indicate mRFP staining in the cytoplasm of parvalbumin negative PCs. Scale bar: 20 µm.

E: Confocal images (maximum projection) of cerebellar slice (lobule VI) from the GRP81-mRFP murine line stained for mRFP (*mRFP*) and NeuN (*NeuN*). Nuclei were stained with the fluorescent dye Hoechst (*Hoechst*). Asterisks indicate GCs. Scale bar: 10 µm.

F: Pie charts showing the percentage of PCs recorded in each cerebellar zone.

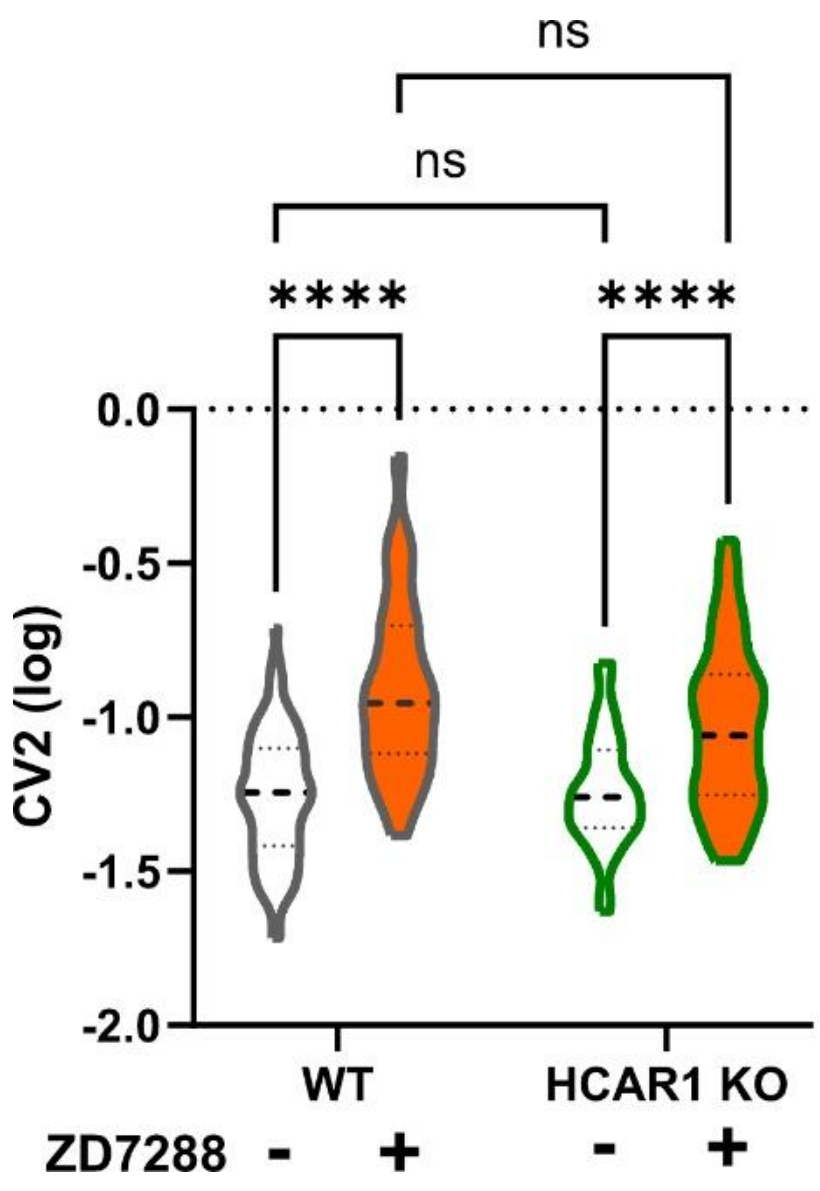

Figure S3: HCN pharmacological block increased firing variability in WT and HCAR1 KO Purkinje cells

Violin plots showing the coefficient of variation 2 (CV2) (logarithmic values) quantified for WT and KO PCs recorded at near-physiological temperature in control condition and in presence of ZD7288 in the bath. Median with the first and third quartile, n.s = P > 0.05, **** = P < 0.0001, Two-Way ANOVA, WT control: N = 3, n = 87, WT ZD7288. N = 4, n = 30, KO control: N = 5, n = 71, KO ZD7288: N = 3, n = 45.

Also see Table S1.

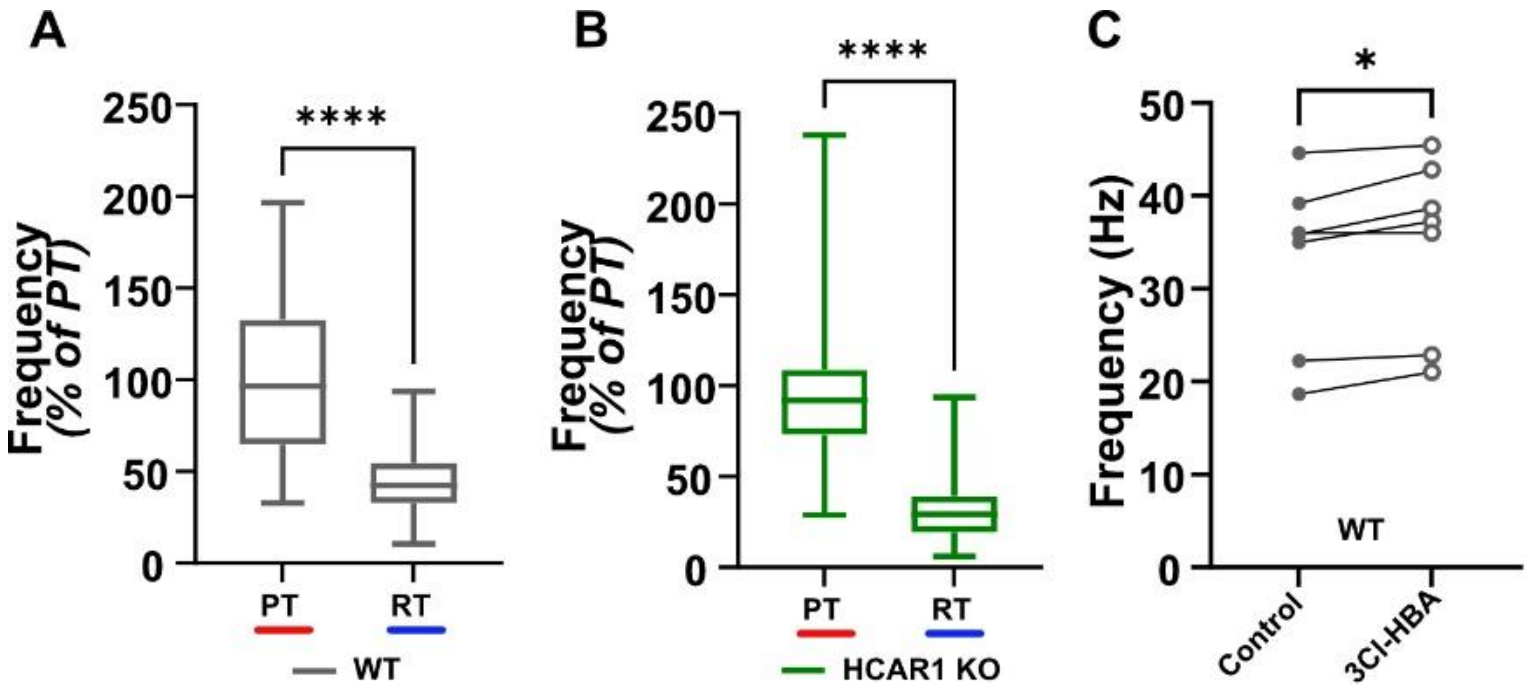

Figure S4: WT and HCAR1 KO Purkinje cells displayed a decreased firing frequency at room temperature

A: Box and whiskers plots showing the firing frequency quantified for WT PCs at near-physiological temperature (PT) and room temperature (RT). Data are normalized to the mean firing frequency obtained at RT. Median, whiskers: minimal and maximal value, **** = $P < 0.0001$, Mann-Whitney test, PT: N = 3, n = 87, RT: N = 3, n = 85.

B: Box and whiskers plots showing the firing frequency quantified for KO PCs at near-physiological temperature (PT) and room temperature (RT). Data are normalized to the mean firing frequency obtained at RT. Median, whiskers: minimal and maximal value, **** = $P < 0.0001$, Mann-Whitney test, PT: N = 5, n = 71, RT: N = 4, n = 153.

C: Before-after plot showing the effect of the non-metabolized HCAR1 agonist 3Cl-HBA on WT PCs firing. * = $P < 0.05$, Paired t-test, N = 2, n = 7.

Also see Table S1.

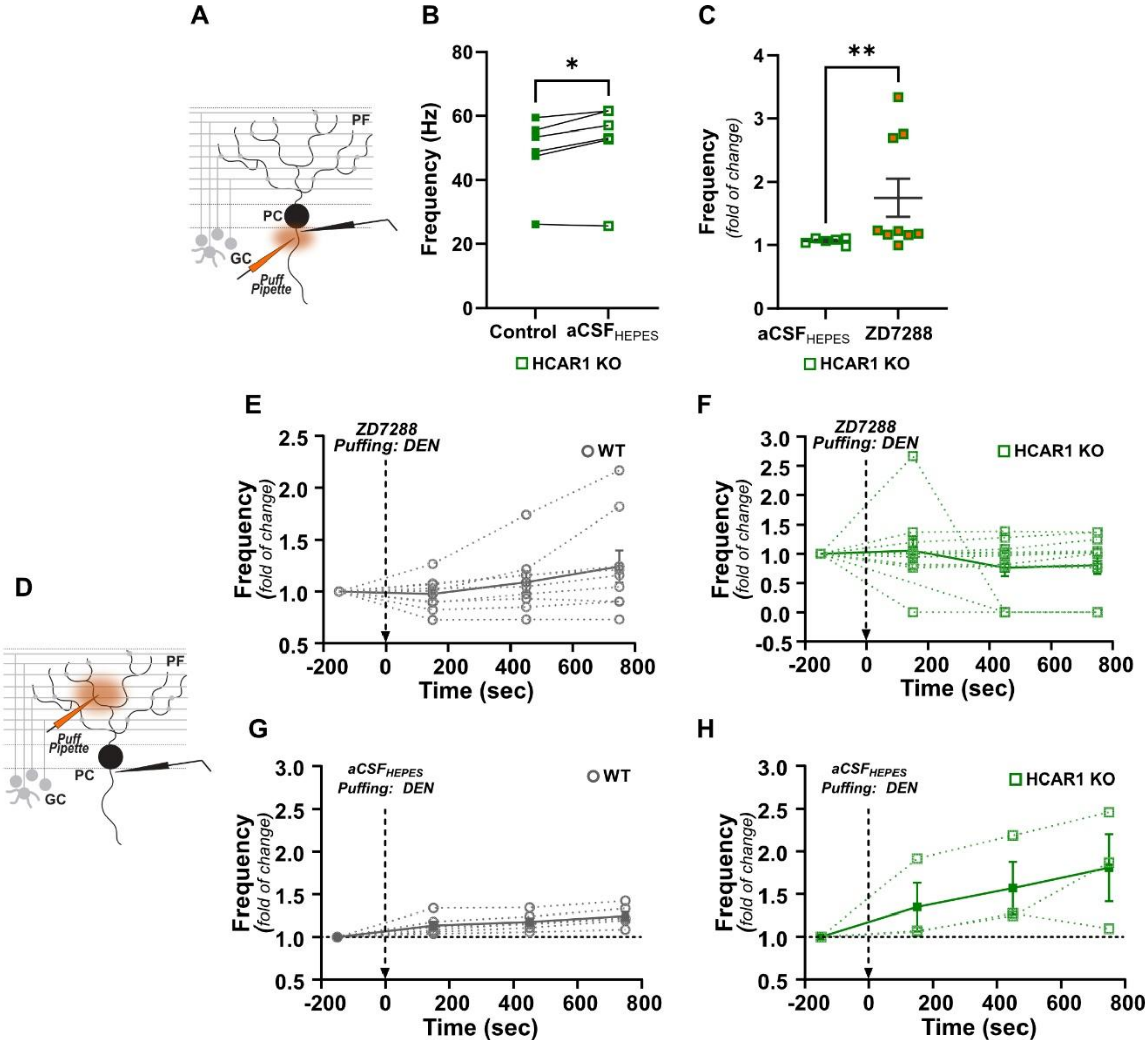

Figure S5: Purkinje cells frequency is sensitive to mechanical stimulation

A: AIS puffing experiment, schematic representation of experimental design .

B: Before-after plots showing HEPES-based aCSF ($aCSF_{HEPES}$)-mediated effect on the frequency of WT PC firing frequency when puffed at the AIS (t = 750 sec). * = P < 0.05, Paired t-test, N = 2, n = 6.

C: Scatter plots showing the more pronounced ZD7288-mediated effect on the frequency of KO PCs when puffed at the AIS (t = 750 sec) in comparison to $aCSF_{HEPES.}$ Each cell was normalized to its frequency under control condition (t = -150 sec). Mean ± SEM, ** = P < 0.01, Mann-Whitney test, aCSF: N = 2, n = 6, ZD7288: N = 4, n = 9.

D: DEN puffing experiment, schematic representation.

E: Scatter plot showing the overtime quantification of the firing frequency of WT PC after ZD7288 puff at the dendrites (light symbols: individual values, dark symbols: mean ± SEM, N = 3, n = 9). Each cell was normalized to its frequency under control condition (t = -150 sec).

F: Scatter plots showing the overtime quantification of the firing frequency of KO PCs after ZD7288 puff at the dendrites (light symbols: individual values, dark symbols: mean ± SEM, N = 4, n = 12). Each cell was normalized to its frequency under control condition (t = -150 sec).

G: Scatter plot showing the overtime quantification of the firing frequency of WT PCs after HEPES-based aCSF ($aCSF_{HEPES}$) puff at the dendrites (light symbols: individual values, dark symbols: mean ± SEM, N = 2, n = 6). Each cell was normalized to its frequency under control condition (t = -150 sec).

H: Scatter plot showing the overtime quantification of the firing frequency of KO PCs after HEPES-based aCSF ($aCSF_{HEPES}$) puff at the dendrites (light symbols: individual values, dark symbols: mean ± SEM, N = 1, n = 3). Each cell was normalized to its frequency under control condition (t = -150 sec).

Also see Table S1.

Table S1: Statistical tests

**Figure, panel**	**Statistical Test**	**P value**	**N, n**
**Figure 1**			
Figure 1, **B**	Multiple linear regression	0.021	WT, N = 17<br>HCAR1 KO: N = 19
Figure 1, **C**	Mann Whitney	0.01	WT, N = 17<br>HCAR1 KO:N = 19
Figure 1, **D**	Unpaired t-test	0.11	WT: N = 17<br>HCAR1 KO: N = 19
Figure 1, **H**	Two-way RM ANOVA with Tukey's multiple comparison test	WT<br>Day 1 vs Day 2<br>< 0.0001	WT: N =34<br>HCAR1 KO: N = 9
Figure 1, **H**	Two-way RM ANOVA with Tukey's multiple comparison test	WT<br>Day 1 vs Day 3<br>< 0.0001	WT: N =34<br>HCAR1 KO: N = 9
Figure 1, **H**	Two-way RM ANOVA with Tukey's multiple comparison test	WT<br>Day 2 vs Day 3<br>0.45	WT: N =34<br>HCAR1 KO: N = 9
Figure 1, **H**	Two-way RM ANOVA with Tukey's multiple comparison test	HCAR1 KO<br>Day 1 vs Day 2<br>0.99	WT: N =34<br>HCAR1 KO: N = 9
Figure 1, **H**	Two-way RM ANOVA with Tukey's multiple comparison test	HCAR1 KO<br>Day 1 vs Day 3<br>0.67	WT: N =34<br>HCAR1 KO: N = 9
Figure 1, **H**	Two-way RM ANOVA with Tukey's multiple comparison test	HCAR1 KO<br>Day 2 vs Day 3<br>0.75	WT, N =34<br>HCAR1 KO: N = 9
Figure 1, **H**	Two-way RM ANOVA with Tukey's multiple comparison test	WT vs HCAR1 KO<br>Day 1<br>0.57	WT: N =34<br>HCAR1 KO, N = 9
Figure 1, **H**	Two-way RM ANOVA with Tukey's multiple comparison test	WT vs HCAR1 KO<br>Day 2<br>0.013	WT: N =34<br>HCAR1 KO: N = 9
Figure 1, **H**	Two-way RM ANOVA with Tukey's multiple comparison test	WT vs HCAR1 KO<br>Day 3<br>0.016	WT: N =34<br>HCAR1 KO: N = 9
**Figure 2**			
Figure 2, **C**	Two-Way ANOVA with Sidak's multiple comparison test	WT vs HCAR1 KO<br>LIII<br>0.97	WT: N = 3<br>HCAR1 KO: N = 3
Figure 2, **C**	Two-Way ANOVA with Sidak's multiple comparison test	WT vs HCAR1 KO<br>LVI<br>0.62	WT: N = 3<br>HCAR1 KO: N = 3
Figure 2, **C**	Two-Way ANOVA with Sidak's multiple comparison test	WT vs HCAR1 KO<br>LVIII<br>0.86	WT: N = 3<br>HCAR1 KO: N = 3
Figure 2, E ***left***	Mann-Whitney test	0.023	WT: N = 3, n = 87<br>HCAR1 KO: N = 5, n = 71
Figure 2, E ***righ*****t**	Mann-Whitney test	0.5	WT: N = 3, n = 87

			HCAR1 KO: N = 5, n = 71
**Figure 3**			
Figure 3, **B**	Two-Way ANOVA with Holm-Sidak post hoc test	WT<br>control vs ZD7288<br>0.1	WT, control: N = 3, n = 87<br>WT, ZD7288: N = 4, n = 30
Figure 3, **B**	Two-Way ANOVA with Holm-Sidak post hoc test	HCAR1 KO<br>control vs ZD7288<br>< 0.0001	HCAR1, control: N = 5, n = 71<br>HCAR1 KO, ZD7288: N = 3, n = 45
Figure 3, **B**	Two-Way ANOVA with Holm-Sidak post hoc test	WT vs HCAR1 KO control<br>0.038	WT, control: N = 3, n = 87<br>HCAR1, control: N = 5, n = 71
Figure 3, **B**	Two-Way ANOVA with Holm-Sidak post hoc test	WT vs HCAR1 KO ZD7288<br>0.75	WT, ZD7288: N = 4, n = 30<br>HCAR1 KO, ZD7288: N = 3, n = 45
Figure 3, **E**	Mann-Whitney test	0.0055	WT: N = 3, n = 9<br>HCAR1 KO: N = 3, n = 8
**Figure 4**			
Figure 4, **A**	Mann-Whitney test	< 0.0001	WT: N = 3 mice, n = 85<br>HCAR1 KO: N = 4, n = 153
Figure 4, **B *left***	Mann-Whitney test	0.0005	WT: N = 3 mice, n = 85<br>HCAR1 KO: N = 4, n = 153
Figure 4, **B *right***	Mann-Whitney test	0.0023	WT: N = 3 mice, n = 85<br>HCAR1 KO: N = 4, n = 153
**Figure 5**			
Figure 5, **B *left***	Paired t-test	0.895	WT: N = 4, n = 10
Figure 5, **B *right***	Wilcoxon matched-pairs signed rank test	0.0078	HCAR1 KO: N = 4, n = 9
Figure 5, **C *center***	Paired t-test	0.111	WT: N = 2, n = 3
Figure 5, C ***right***	Paired t-test	0.045	HCAR1 KO: N = 3, n = 5
**Figure S1**			
Figure S1, **A**	Unpaired t-test	0.0014	WT: N = 17<br>HCAR1 KO = 19
Figure S1, **B**	Unpaired t-test	0.032	WT: N = 17<br>HCAR1 KO = 19
Figure S1, **C**	Unpaired t-test	0.0003	WT: N = 17<br>HCAR1 KO = 19

Figure S1, **D**	Two-way ANOVA, Sidak's multiple comparisons test;	RF: 0.0021<br>RH: 0.0023<br>LF: 0.0003<br>LH: 0.0013	WT: N = 17<br>HCAR1 KO = 19
Figure S1, **E**	Two-way ANOVA, Sidak's multiple comparisons test;	RF: 0.0009<br>RH: 0.0025<br>LF: 0.0001<br>LH: 0.0011	WT: N = 17<br>HCAR1 KO = 19
Figure S1, **H**	Fisher's test	< 0.0001	WT: N = 41<br>HCAR1 KO: N = 29
Figure S1, **I**	Unpaired t-test	0.61	WT: N = 32<br>HCAR1 KO: N = 9
**Figure S3**			
Figure S3	Two-Way ANOVA with Holm-Sidak post hoc test	WT<br>control vs ZD7288<br>< 0.0001	WT, control: N = 3, n = 87<br>WT, ZD7288: N = 4, n = 30
Figure S3	Two-Way ANOVA with Holm-Sidak post hoc test	HCAR1 KO<br>control vs ZD7288<br>< 0.0001	HCAR1, control: N = 5, n = 71<br>HCAR1 KO, ZD7288: N = 3, n = 45
Figure S3	Two-Way ANOVA with Holm-Sidak post hoc test	WT vs HCAR1 KO control<br>0.693	WT, control: N = 3, n = 87<br>HCAR1, control: N = 5, n = 71
Figure S3	Two-Way ANOVA with Holm-Sidak post hoc test	WT vs HCAR1 KO ZD7288<br>0.058	WT, ZD7288: N = 4, n = 30<br>HCAR1 KO, ZD7288: N = 3, n = 45
**Figure S4**			
Figure S4, **A**	Mann-Whitney test	< 0.0001	*PT,* WT: N = 3, n = 87<br>*RT,* WT: N = 3 ,n = 85
Figure S4, **B**	Mann-Whitney test	< 0.0001	*PT*, HCAR1 KO: N = 5, n = 71<br>*RT,* HCAR1 KO: N = 4 ,n = 153
Figure S4, **C**	Paired t-test	0.011	N = 2, n = 7
**Figure S5**			
Figure S5, **B**	Paired t-test	0.016	HCAR1 KO: N = 2, n = 6
Figure S5, **C**	Mann-Whitney test	0.0076	aCSF: N = 2, n = 6<br>ZD7288: N = 4, n = 9

Bibliography

S1. Buscemi, L., Price, M., Castillo-Gonzalez, J., Chatton, J.Y., and Hirt, L. (2022). Lactate Neuroprotection against Transient Ischemic Brain Injury in Mice Appears Independent of HCAR1 Activation. Metabolites *12*. 10.3390/metabo12050465.
